# Preliminary Study on Sexual Maturation Pattern of *Shenxian* Pigs and Molecular Characteristics of Sexual Precocity in Boars

**DOI:** 10.3390/ijms27041663

**Published:** 2026-02-09

**Authors:** Jialong Zhao, Shan Yang, Haitao Chen, Yu Li, Jiahui Yuan, Mingxin Sun, Chunlian Lu, Hongzhan Cao

**Affiliations:** College of Animal Science and Technology, Hebei Agricultural University, Baoding 071000, China; 13293011030@163.com (J.Z.); yangshan1123036@163.com (S.Y.); 13931938488@163.com (H.C.); ly18731456994@163.com (Y.L.); 19931966038@163.com (J.Y.); 19932747941@163.com (M.S.)

**Keywords:** *Shenxian* pig, sexual precocity, production performance, testis, transcriptome sequencing

## Abstract

This study aimed to determine the sexual maturation pattern of *Shenxian* pigs by combining observation, teaser boar testing, and back-pressure methods, and to apply this pattern for early breeding to shorten the generation interval and increase production efficiency. Subsequently, high-throughput transcriptome technology was used to compare gene expression levels in testicular tissues of *Shenxian* pigs before and after sexual maturity, as well as between sexually mature *Shenxian* pigs and *Shenxian* × Large White crossbred pigs. Functional analysis of differentially expressed genes (DEGs) was conducted to screen candidate genes related to sexual maturation and precocity in *Shenxian* pigs. The results showed that boars reached sexual maturity at an average age of 116 days in winter and 129 days in summer. For sows, the first estrus occurred at 114 days, the second at 134 days, and the third at 154 days in winter; corresponding ages in summer were 125, 144, and 164 days, respectively. The duration of estrus was around 3 days, and the estrus interval was approximately 20 days for both seasons. Comparative trials revealed no significant change in production performance when selection and first mating were conducted at 5 months of age compared to previous practices. Transcriptome sequencing of testicular tissues before and after sexual maturity in *Shenxian* pigs identified 6016 upregulated genes, primarily associated with reproduction and sperm function, influencing sexual maturation. The comparison between sexually mature *Shenxian* pigs and crossbred pigs identified 582 upregulated genes, mainly involved in hormone synthesis, affecting the onset of puberty in *Shenxian* pigs. After intersecting and functionally analyzing the upregulated genes from both sets, SRD5A1 and CYP11B2 were selected as the most likely candidate genes to affect precocious puberty in *Shenxian* pigs.

## 1. Introduction

The *Shenxian* pig is a local pig breed from Hebei Province, China, characterized by its strong adaptability, high disease resistance, early sexual maturity, and excellent meat quality [1]. Boars can ejaculate as early as three months of age, while sows can conceive and give birth at four months of age, making it a typical early-maturing breed. Under specific management practices, early sexual maturity can shorten the generation interval of sows, enabling faster breeding. Given the scarcity of *Shenxian* pigs, utilizing this trait can help cultivate more individuals of the breed and facilitate crossbreeding efforts to develop new varieties. As a distinctive local breed, studying the patterns and molecular mechanisms of early sexual maturity in *Shenxian* pigs can not only improve their rearing but also allow them to serve as parental stock in hybridization, passing on their superior genetic traits.

Previous studies have shown that the age at first mating is a critical factor affecting the litter performance of sows. Mating too early can lead to miscarriages and reproductive organ damage in sows, while mating too late can impair reproductive performance and make mating difficult [2]. Cottney [3] suggested that the third estrus cycle is optimal for mating sows, a standard still followed in the production of introduced breeds. Whether this mating model is suitable for *Shenxian* pigs warrants investigation. Prior research on the estrus stages and cycles of pigs has divided boar sexual behavior into mounting, penis extension, and ejaculation stages based on hormonal changes, while sows are recorded according to different estrus cycles, noting estrus intervals and duration [4,5]. The same methods will be applied to study the estrus cycles of *Shenxian* pigs.

Therefore, this experiment aims to determine the sexual maturity patterns of *Shenxian* pigs by combining observational methods, estrus detection, and back pressure tests, comparing the differences in sexual maturity between winter and summer, and identifying scientifically sound selection times and indicators for the application of early sexual maturity through performance testing. Due to conservation constraints, ovarian tissues from *Shenxian* sows were not collected, so this experiment focuses only on the genetic exploration of early sexual maturity in boars. We will employ transcriptome sequencing, first analyzing testicular tissues of *Shenxian* pigs before and after sexual maturity (post-maturity group labeled S1, pre-maturity group labeled S2) to identify differentially expressed genes and screen for candidate genes related to sexual maturity. Subsequently, transcriptome sequencing will be conducted on testicular tissues of sexually mature *Shenxian* pigs (labeled S group) and their crossbreeds with *Yorkshire* pigs (labeled YS group) to analyze differentially expressed genes and identify genes specifically expressed in sexually mature *Shenxian* pigs. By integrating the results of the two transcriptome sequencing experiments, overlapping genes will be analyzed for their functions, and candidate genes potentially influencing the early sexual maturity of *Shenxian* pigs will be screened based on gene functions.

## 2. Results

### 2.1. Determination of Sexual Maturation Pattern and Selection/Mating Time for Shenxian Pigs

The sexual maturation timing and patterns of male and female *Shenxian* pigs were determined through observational methods, estrus detection, and back pressure tests. The boars were categorized into three stages: mounting, penile extension, and ejaculation. Mounting behavior indicates that the pig is already stimulated by sex hormones, while ejaculation behavior signifies that sex hormone stimulation has peaked, enabling the completion of the entire sexual act. The sows were divided into three stages based on their estrus cycles: the first, second, and third estrus cycles. Since significant seasonal temperature variations can also affect the sexual maturation of pigs, this experiment conducted statistics for different seasons, specifically winter and summer.

As shown in Table 1, the average age at sexual maturity for male *Shenxian* pigs was 116 days in winter and 129 days in summer. After comparison, it was found that there was no significant difference in the timing of mounting behavior between winter and summer (*p* > 0.05). However, the timing of penile extension and ejaculation occurred significantly earlier in winter than in summer (*p* < 0.01), indicating that sexual maturation occurs significantly earlier in low-temperature seasons compared to high-temperature seasons.

As shown in Table 2, the first estrus cycle of female *Shenxian* pigs occurred at 114 days of age in winter, the second at 134 days, and the third at 154 days. In summer, the first estrus cycle occurred at 125 days of age, the second at 144 days, and the third at 164 days. The duration of estrus was approximately 3 days in both winter and summer, with an estrus interval of around 20 days. Across the three estrus cycles, the onset and conclusion of estrus occurred significantly earlier in winter than in summer (*p* < 0.01). Regarding the duration of estrus, it was significantly longer in winter than in summer during the first estrus cycle (*p* < 0.01), showed no significant difference in the second estrus cycle (*p* > 0.1), and exhibited a trend of being significantly shorter in summer during the third estrus cycle (*p* < 0.1). In terms of estrus intervals, the interval during the second estrus cycle was significantly longer in winter than in summer (*p* < 0.01). Overall, the timing of estrus was significantly earlier in low-temperature seasons compared to high-temperature seasons.

Since *Shenxian* pigs are bred through natural mating, the reproductive performance of boars is difficult to assess with specific data. Therefore, this experiment only evaluated the reproductive performance of sows, primarily focusing on litter performance. Among the sows with complete data observed in winter, those in their third estrus cycle were selected for mating. After farrowing, data on litter size, birth weight, weaning count, and weaning weight were recorded and compared with the reproductive performance of sows mated after the third estrus cycle during the same period to determine the feasibility of mating during the third estrus cycle. A total of 20 sows in their third estrus cycle were selected for first mating and designated as the experimental group, labeled C1. Additionally, 20 sows mated after the third estrus cycle during the same period were selected as the control group, labeled C2. The litter performance of the C1 and C2 groups was compared to evaluate the feasibility of mating *Shenxian* sows during the third estrus cycle.

As shown in Table 3, the C1 group exhibited a trend of reduced litter size, though the difference was not statistically significant (*p* < 0.1). No significant differences were observed in birth weight, weaning count, weaning weight, or survival rate (*p* > 0.1). Therefore, mating *Shenxian* pigs during the third estrus cycle does not significantly impact their reproductive performance.

The above experiments revealed that male *Shenxian* pigs are capable of ejaculation at an average age of 4–5 months, while female sows enter their third estrus cycle at an average age of 4.5–5.5 months. Therefore, the selection and breeding timing for *Shenxian* pigs can be adjusted to 5 months of age for boars and the third estrus cycle for sows. Apart from age and estrus cycle, breeding should also take into account individual body size. In previous production practices, the selection and breeding of *Shenxian* pigs primarily considered body weight and backfat thickness. Accordingly, statistical analysis was conducted at the end of the summer observation period during the breeding selection process, with specific results as follows.

Based on the statistical data of body weight and backfat thickness of boars and sows in Table 4, as well as the observed patterns of sexual maturity described above, and considering practical production conditions, the selection criteria for *Shenxian* pigs are set as follows: breeding age at 5 months, body weight over 60 kg, backfat thickness over 16 mm, and mating of sows during their third estrus cycle.

### 2.2. Transcriptome Sequencing Analysis of Testicular Tissues from Shenxian Boars Before and After Sexual Maturity

Three sexually mature boars of the same age and body weight but from different litters were randomly selected from those not chosen for breeding purposes. Similarly, three newly weaned boars of the same age, body weight, and from different litters were selected. The immature boars were designated as the control group (S2), while the sexually mature boars served as the experimental group (S1). Testicular tissues were collected via physical castration. The tissues were bisected along the midline, and pea-sized samples were taken from the central region. After rinsing with PBS buffer, the samples were placed in cryovials and rapidly frozen in liquid nitrogen for preservation. They were then transported to the laboratory for RNA extraction and transcriptome sequencing.

First, RNA was extracted, and its concentration was measured. The integrity of the RNA was assessed via gel electrophoresis. Samples that passed quality control were used for library construction and subsequent sequencing analysis.

As shown in Table 5, a total of 43.4 Gb of clean bases were obtained from the transcriptome sequencing, with Q30 values ranging from 92.86% to 94.43% and GC content between 46.18% and 48.43%. As indicated in Table 6, alignment of the clean reads with the reference genome revealed a mapping rate exceeding 95% for each sample, with the accuracy of clean reads ranging from 91.48% to 92.37%. The clean bases obtained after filtering raw bases were no less than 6.64 Gb, with Q20 > 97.49%, Q30 > 92.8%, and GC content > 46.60%, demonstrating high-quality transcriptome sequencing data suitable for subsequent research.

**Figure 1:**
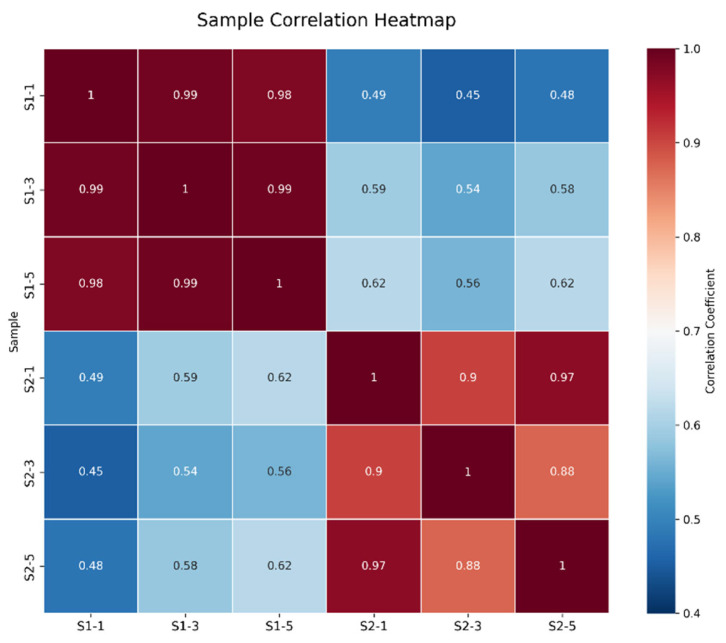
Correlation Analysis Between S1 and S2 Samples.

**Figure 2:**
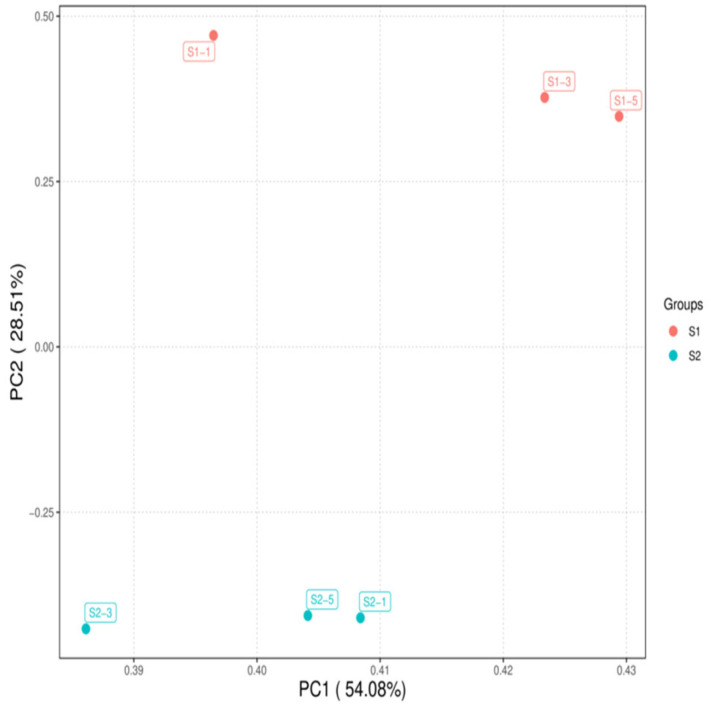
Principal component analysis of samples S1 and S2.

As shown in Figure 1, the correlations among the samples in each group were all significant. As illustrated in Figure 2, the samples within each group clustered together at similar positions, while distinct separation between the two groups was evident. These results met the criteria for further analysis.

**Figure 3:**
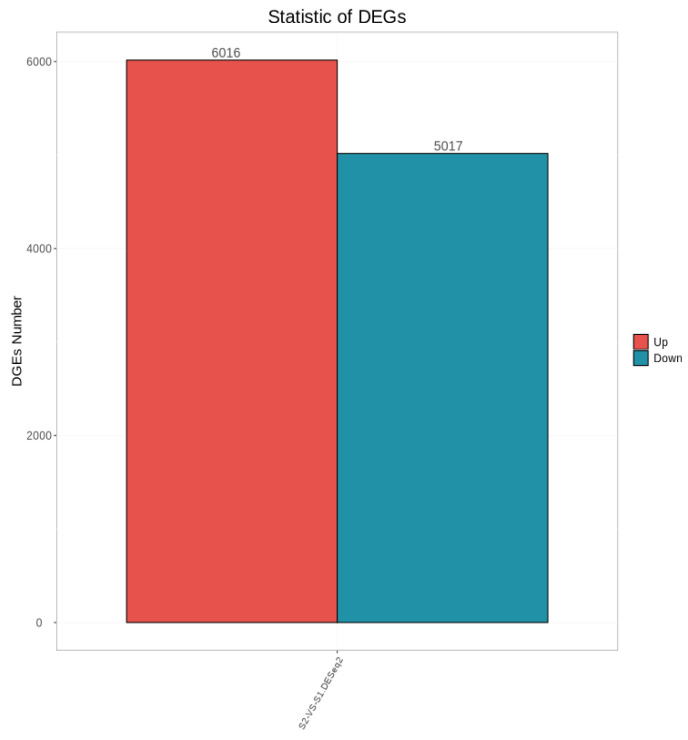
Statistical Plot of Differentially Expressed Genes Between Groups S1 and S2.

**Figure 4:**
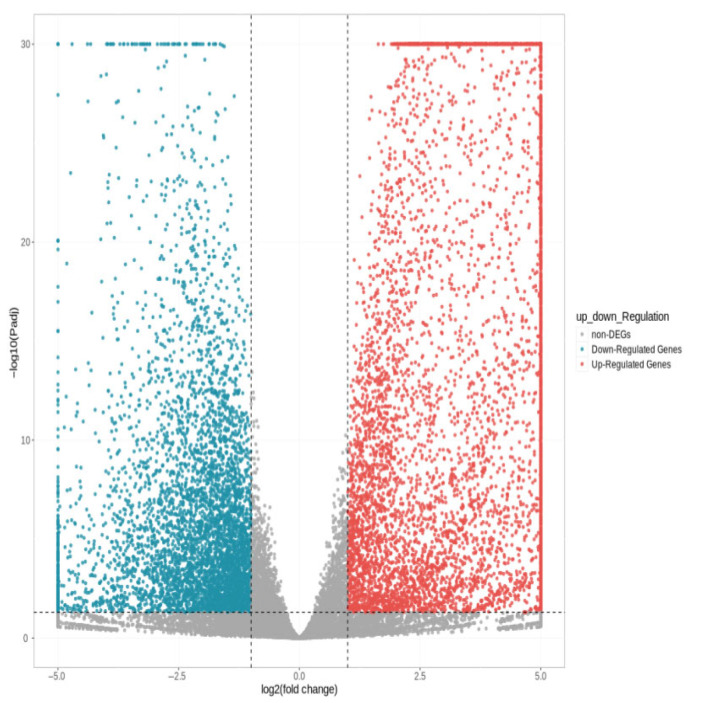
Volcano diagram of differentially expressed genes S1 and S2.

**Figure 5:**
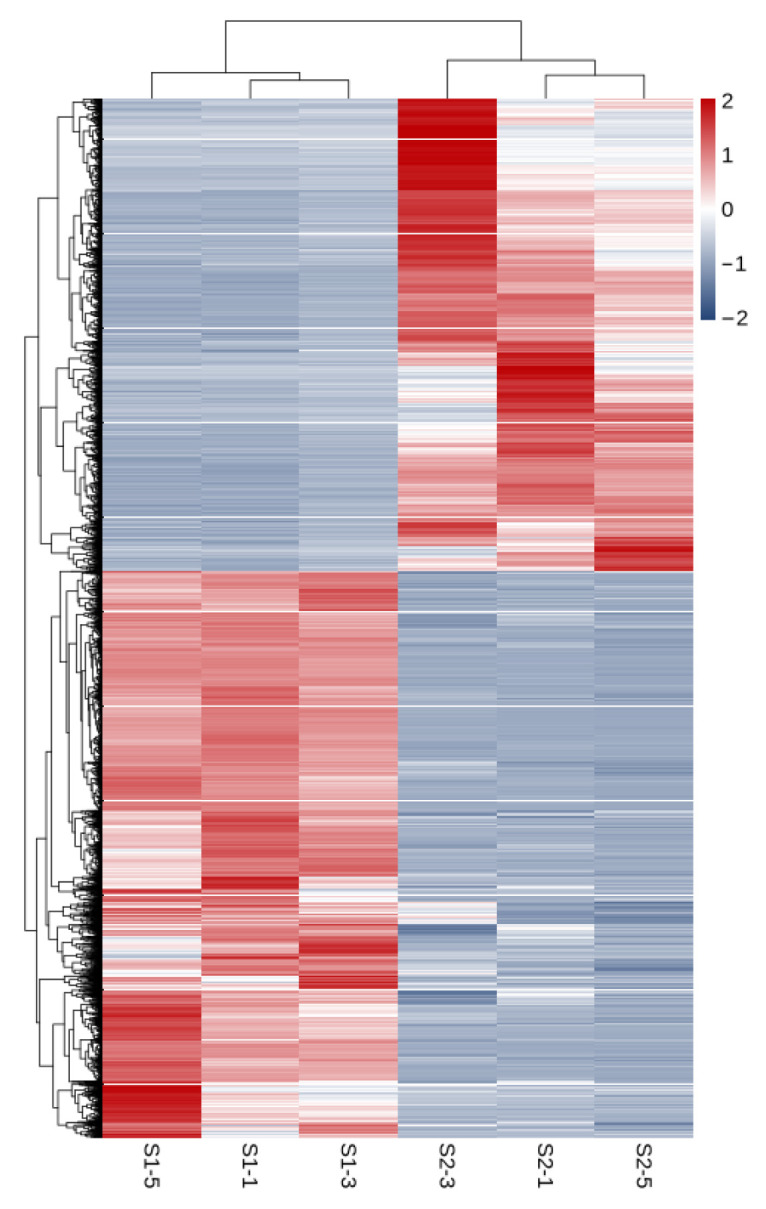
Heat map of S1 and S2 clustering analysis.

As shown in Figure 3, a total of 11,033 differentially expressed genes were screened, among which 6016 were up-regulated and 5017 were down-regulated. The distribution of these differentially expressed genes in the volcano plot is illustrated in Figure 4. The union and intersection of differentially expressed genes were extracted from the multi-group comparison results, and clustering heatmap analysis was performed using the FPKM values of each sample. The results, as shown in Figure 5, revealed distinct differences in gene distribution between groups, while genes within each group were largely similar.

**Figure 6:**
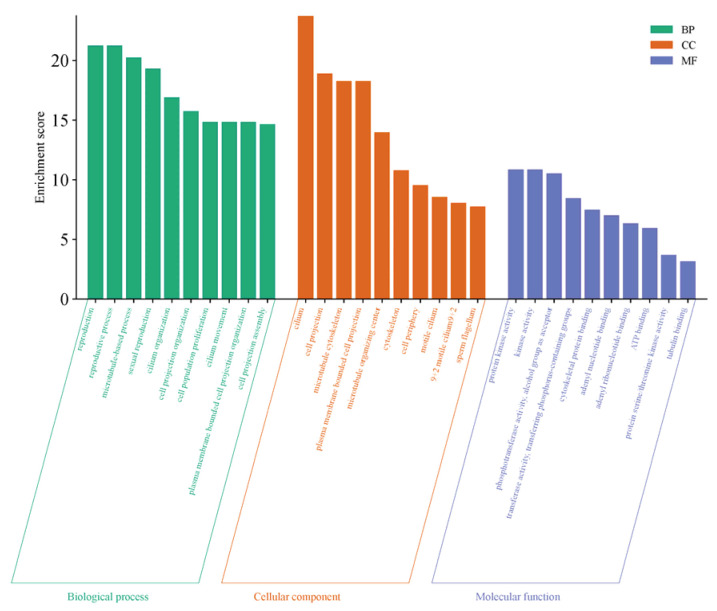
GO Functional Enrichment Plot for S1, S2.

The GO functional enrichment of differentially expressed genes primarily focuses on three categories: Biological Process (BP), Cellular Component (CC), and Molecular Function (MF). The top 10 significantly enriched functional terms in each category were selected to create bar charts, as shown in Figure 6. This gene set exhibits systematic functional enrichment in testicular maturation and spermatogenesis, not only directly participating in core biological processes such as reproduction and sexual reproduction but also being enriched in pathways related to cilia organization and movement, as well as cell population proliferation, which collectively support sperm formation and cell renewal. At the structural level, the genes are highly concentrated in cellular components such as cilia, sperm flagella, and microtubule-related structures, indicating their critical role in the assembly of sperm tail structures and motility apparatus. Meanwhile, the enrichment of molecular functions such as protein kinase activity, ATP binding, and tubulin binding further reveals the underlying kinase signaling transduction, energy metabolism, and cytoskeletal dynamic regulation mechanisms essential for these processes. These findings collectively demonstrate that, at the transcriptional level, this gene set comprehensively supports the cellular structures, proliferation regulation, and signaling networks required for spermatogenesis, flagellar formation, and functional implementation during testicular maturation.

**Figure 7:**
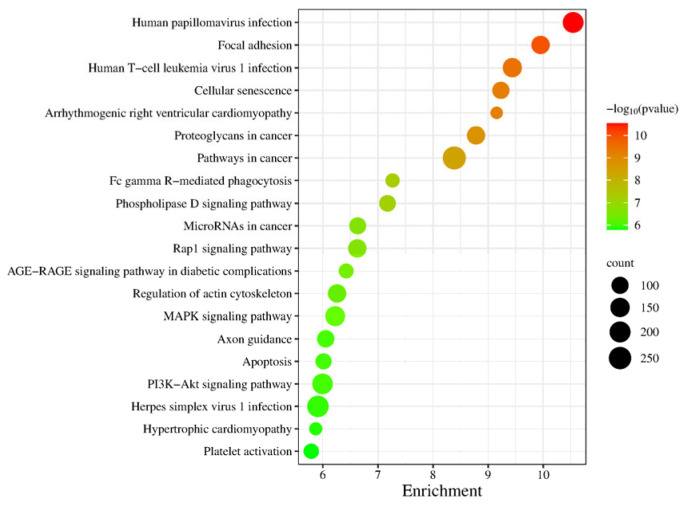
KEGG Pathway Analysis Plot for S1, S2.

In the KEGG signaling pathway analysis of differentially expressed genes, the top 20 significantly enriched pathways were selected to generate a bubble chart, as shown in Figure 7. Enrichment analysis of this gene set at the pathway level revealed that the key signaling networks related to testicular maturation and spermatogenesis mainly include the MAPK signaling pathway, PI3K-Akt signaling pathway, Rap1 signaling pathway, and regulation of the actin cytoskeleton. These pathways collectively regulate the proliferation, differentiation, survival, and structural remodeling of spermatogenic cells. Additionally, the enrichment of the apoptosis pathway reflects the regulatory mechanism of selective cell clearance during spermatogenesis, while the cellular senescence pathway may be involved in maintaining germ cell homeostasis. Although some pathways in the list are associated with viral infections or diseases, others such as focal adhesion, phospholipase D signaling pathway, and axon guidance also play roles in cell adhesion, membrane dynamics, and morphogenesis, indirectly supporting cell migration and structural assembly during spermatogenesis. In summary, these pathways systematically constitute the molecular regulatory foundation for spermatogenesis and testicular functional maturation from multiple levels, including signal transduction, cytoskeletal dynamics, cell cycle, and cell death.

**Figure 8:**
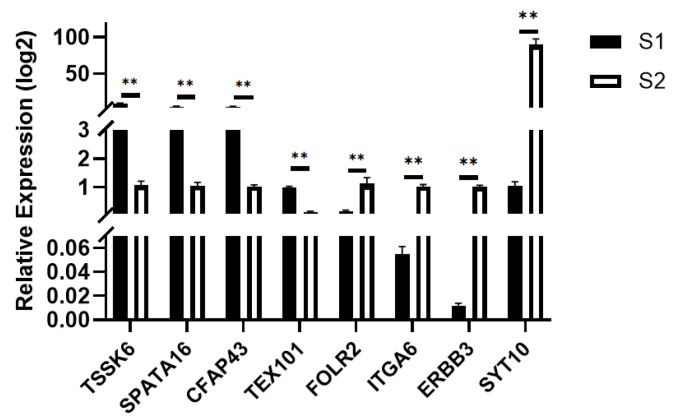
q-PCR Validation for S1, S2. ** *p* < 0.01.

Using the pre-sexual maturity group S2 as the control and the post-sexual maturity group S1 as the experimental group, quantitative real-time PCR (qPCR) validation was performed. The *TPB* gene was used as the internal reference, and the relative expression levels of the target genes were calculated using the 2^−ΔΔCt^ method. The significance was compared with the transcriptome sequencing results, and bar charts were generated for analysis. As shown in Figure 8, the results showed that *TSSK6*, *SAPA16*, *CFAPA43*, and *TEX101* were significantly up-regulated, while *FOLR2*, *ITGA6*, *ERBB3*, and *SYT10* were significantly down-regulated, which was consistent with the transcriptome sequencing results.

### 2.3. Transcriptome Sequencing of Testicular Tissues from Shenxian Pigs and Shenxian × Large White Crossbred Pigs

The differentially expressed genes screened in the above experiments were primarily enriched in pathways related to reproduction and spermiogenesis, with no significant genes identified that directly regulate hormone levels affecting early sexual maturity. Therefore, testicular tissues were collected from three *Shenxian* × Yorkshire hybrid pigs, which were selected during the same period as the experimental pig group mentioned above. The hybrid pigs served as the control group, labeled as the YS group, while the *Shenxian* pigs served as the experimental group, labeled as the S group. RNA was extracted from both groups for transcriptome sequencing.

As shown in Table 7, a total of 39.21 Gb of clean bases were obtained from the transcriptome sequencing, with Q30 values ranging from 97.57% to 97.67% and GC content between 49.8% and 50.47%. As indicated in Table 8, alignment of the clean reads with the reference genome revealed a mapping rate exceeding 97% for each sample, and the accuracy of the clean reads ranged from 94.83% to 95.04%. After filtering the raw bases, the clean bases obtained were no less than 6.14 Gb, with Q20 > 99.36%, Q30 > 97.57%, and GC content > 49.80%, indicating high-quality transcriptome sequencing data suitable for subsequent research.

**Figure 9:**
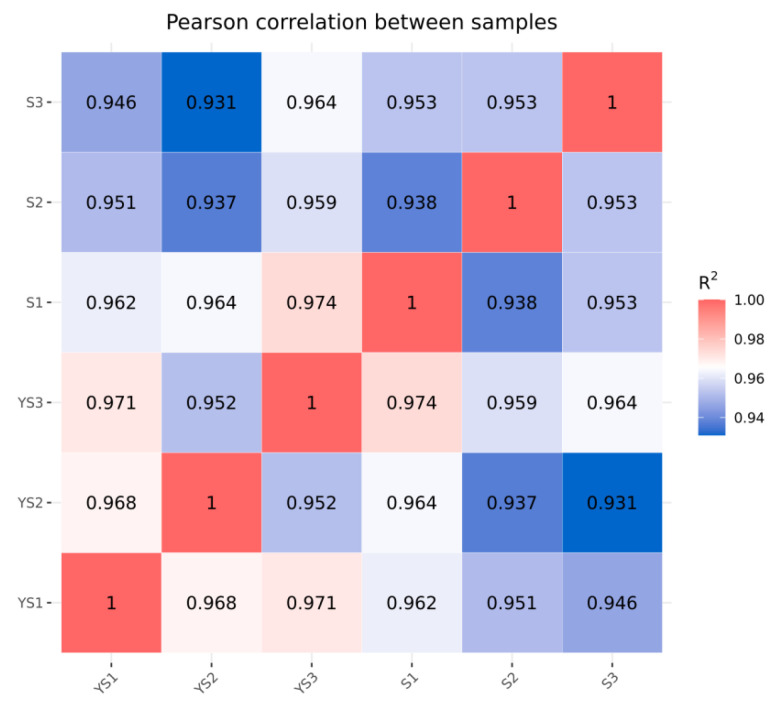
Correlation analysis between YS and S samples.

**Figure 10:**
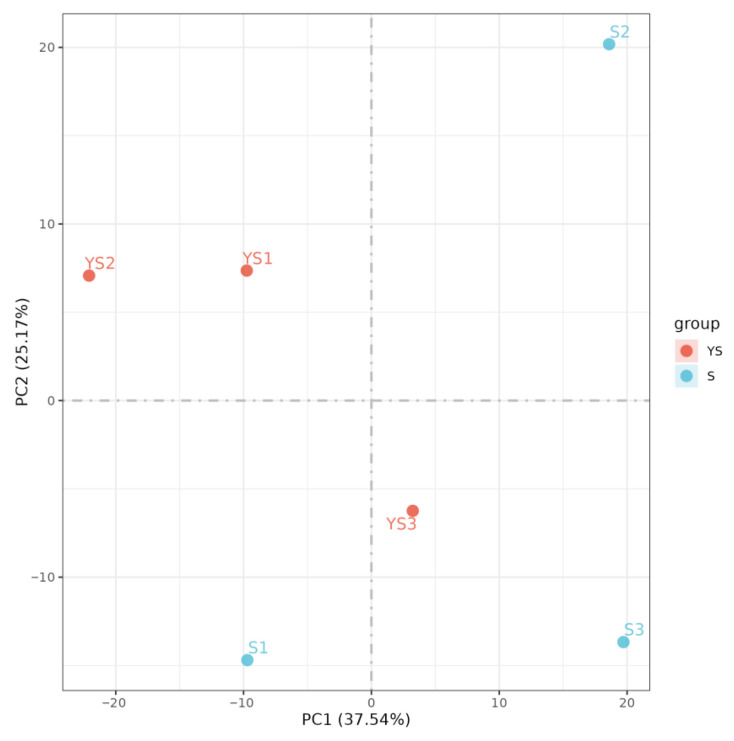
PCA analysis between samples.

As illustrated in Figure 9, the correlations among samples within each group were all statistically significant. In Figure 10, samples within each group clustered together, while clear separation was observed between groups, indicating that the data met the criteria for subsequent analysis.

**Figure 11:**
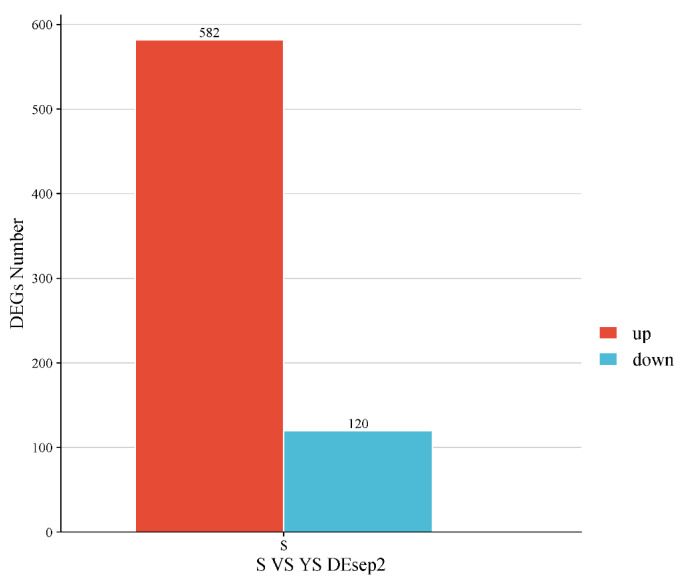
YS, S differentially expressed gene statistics.

**Figure 12:**
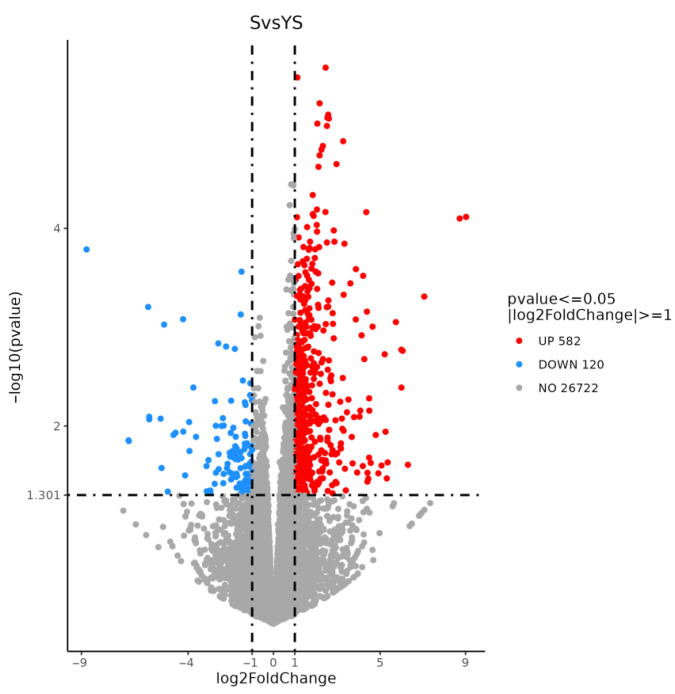
YS, S volcano map of differentially expressed genes.

**Figure 13:**
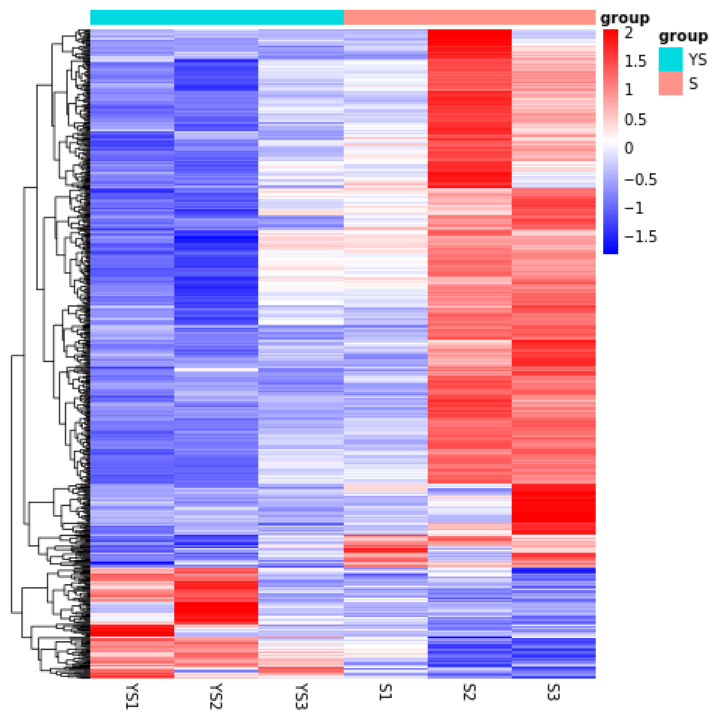
YS, S differential expression gene clustering heatmap.

As shown in Figure 11, a total of 702 differentially expressed genes were screened, among which 582 were up-regulated and 120 were down-regulated. The distribution of these differentially expressed genes is presented in the volcano plot in Figure 12. As illustrated in Figure 13, distinct differences in gene distribution were observed between groups, while genes within each group remained largely similar.

**Figure 14:**
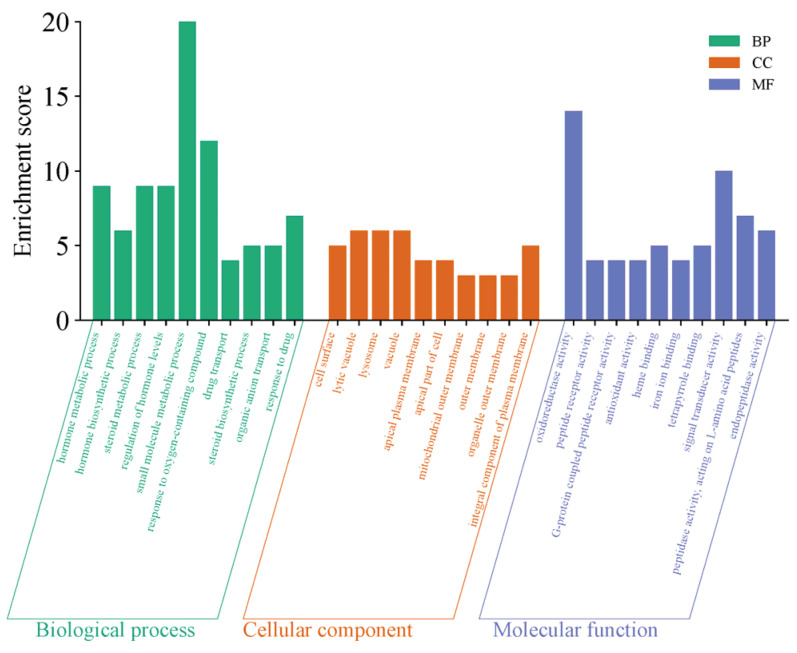
YS, Group S GO functional enrichment map.

The GO functional enrichment of the differentially expressed genes primarily focuses on three categories: Biological Process (BP), Cellular Component (CC), and Molecular Function (MF). The top 10 significantly enriched functional terms from each category were selected to create bar charts, as shown in Figure 14. The functional enrichment characteristics of this gene set are highly consistent with the regulation of sexual maturation, with its core focus directed toward the synthesis, secretion, and signaling networks of sex hormones. In biological processes, the genes are significantly enriched in steroid biosynthesis, hormone metabolism, and hormone level regulation, directly linking to the production and homeostasis maintenance of key steroid hormones such as testosterone and estrogen, which drive sexual maturation. In molecular functions, the enrichment of oxidoreductase activity and heme binding provides the catalytic foundation for the cytochrome P450 enzyme system, which is essential for steroid hormone synthesis, while G protein-coupled peptide receptor activity suggests responsiveness to peptide signaling molecules such as gonadotropins. Together, these functions constitute critical components of hormone synthesis and reception. Additionally, the enrichment in cellular components such as the cell surface, apical plasma membrane, and lysosomes further reflects the cellular basis for transmembrane transport, polarized secretion, and metabolic recycling of hormone precursors. These functions systematically outline an endocrine regulatory program that supports gonad activation, secondary sexual characteristic development, and the initiation of reproductive functions, spanning hormone biosynthesis, oxidative modification, signal recognition, and subcellular localization.

**Figure 15:**
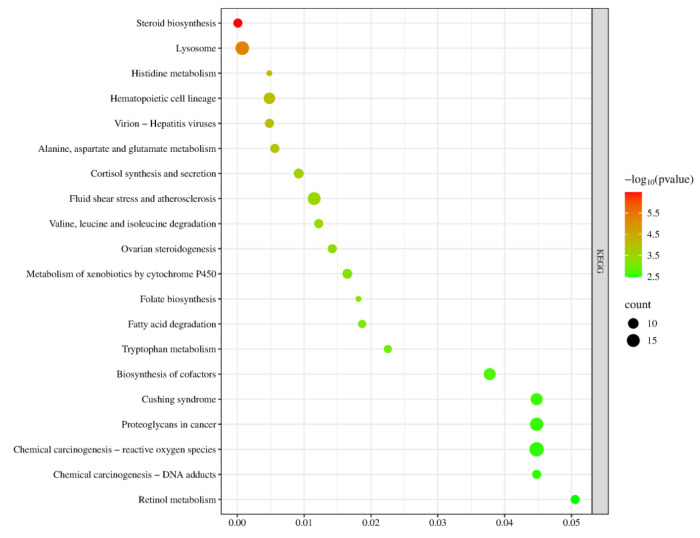
KEGG Pathway Analysis Plot for YS, S Groups.

In the KEGG signaling pathway analysis of the differentially expressed genes, the top 20 significantly enriched pathways were selected to generate a bubble chart. As shown in Figure 15, the pathway enrichment analysis results of this gene set are highly consistent with the core regulatory mechanisms of sexual maturation, primarily focusing on the synthesis and metabolic networks of gonadal steroid hormones. Key pathways such as “ovarian steroidogenesis” and “steroid biosynthesis” directly point to the biosynthesis of sex hormones—the critical event driving sexual maturation. Meanwhile, the enrichment of the “cortisol synthesis and secretion” pathway suggests potential cross-regulation between the hypothalamic–pituitary–gonadal axis and the stress axis, collectively influencing the timing of puberty initiation. To support efficient hormone synthesis, the genes are also enriched in multiple supporting metabolic pathways, including alanine, aspartate, and glutamate metabolism; valine, leucine, and isoleucine degradation; and fatty acid degradation, which provide energy and precursor substrates through catabolism. Additionally, cofactor biosynthesis and retinol metabolism supply essential coenzymes and vitamin A derivatives, such as those required for relevant synthases like cytochrome P450 enzymes. In summary, these pathways systematically constitute a comprehensive metabolic and signaling foundation that supports gonad activation and the development of secondary sexual characteristics, spanning multiple levels from core hormone synthesis, substrate and energy supply, cofactor support, to cellular environmental adaptation.

**Figure 16:**
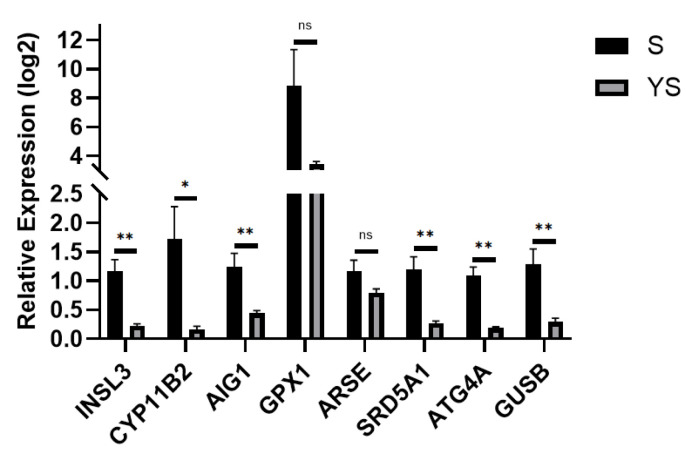
q-PCR Validation for S, YS. * *p* < 0.05, ** *p* < 0.01, ns: not significant.

Using the hybrid pig YS group as the control and the *Shenxian* pig S group as the experimental group, quantitative real-time PCR (qPCR) validation was performed. The *TPB* gene was used as the internal reference, and the relative expression levels of the target genes were calculated using the 2^−ΔΔCt^ method. As shown in Figure 16, the results revealed that *INSL3*, *CYP11B2*, *SRD5A1*, *ARSE*, *AIG1*, *GPX1*, *ATG4A*, and *GUSB* were all up-regulated, which is consistent with the transcriptome sequencing results, thereby validating the accuracy of the transcriptome sequencing data.

### 2.4. Genetic Analysis That May Affect Early Puberty in Pigs in Shenxian County

In the results of the first transcriptome sequencing, most of the screened genes were related to sperm function in *Shenxian* pigs, while genes associated with early sexual maturity showed less pronounced expression. In the second sequencing, it became evident that most of the screened genes were related to hormonal regulation, which may influence the early sexual maturity of *Shenxian* pigs. Therefore, by integrating the results of both sequencing analyses and examining all upregulated genes, it was found that a total of 39 differentially expressed genes were significantly upregulated in *Shenxian* pigs. The names and functions of all these genes are listed in Table 9.

By querying the NCBI database to investigate the functions of these 39 genes, it was discovered that 32 of them have well-documented functions. Based on the results of GO functional enrichment and KEGG pathway analyses, as well as relevant literature reports, the functions of these 32 genes were analyzed. Screen out two genes that are most likely to affect precocious puberty, namely *SRD5A1* and LOC110260194 (i.e., *CYP11B2*). The primary function of *SRD5A1* is to catalyze the conversion of testosterone to dihydrotestosterone. Early accumulation of dihydrotestosterone can promote the premature appearance of secondary sexual characteristics, leading to early sexual maturity. *CYP11B2* belongs to the CYP family, which is involved in the synthesis of sex hormones. *CYP11B2* itself plays a role in the synthesis of adrenal cortex hormones and may indirectly regulate the timing of puberty initiation by influencing hormonal balance in the body.

## 3. Discussion

### 3.1. Sexual Maturation Pattern of Shenxian Pigs

Existing research has revealed differences in the age of sexual maturity between indigenous Chinese pig breeds and foreign breeds. For commonly introduced foreign breeds, the age of sexual maturity for *Yorkshire* pigs is typically 210–240 days, for Landrace boars it is around 210 days, and for *Duroc* pigs it ranges from 210 to 240 days [6]. Among Chinese local breeds, *Rongchang* pigs reach sexual maturity at 3 months for boars and 4 months for sows [7]; *Neijiang* pigs mature at about 110 days for boars and 120 days for sows [8]; and *Jinhua* pigs achieve sexual maturity at 98–102 days for boars and 110–115 days for sows [9]. In this study, the average age of sexual maturity for *Shenxian* pigs was around 4 months, also classifying them as an early-maturing breed.

In this study, both boars and sows reached sexual maturity earlier in winter than in summer, indicating that season has a significant impact on sexual maturation. High summer temperatures, combined with the underdeveloped sweat glands of pigs, can easily induce heat stress. This stress reduces ovulation rates and conception rates in sows, while high temperatures can also affect testosterone production in boars, thereby delaying sexual maturity [10]. Since the *Shenxian* pig pens are equipped with exercise yards that ensure natural light exposure daily, no dedicated lighting equipment was installed in the pens. Therefore, seasonal variation is one of the primary objective factors influencing the sexual maturity of *Shenxian* pigs. During observation, it was also noted that *Shenxian* pigs were significantly more active in summer than in winter. The reason is that shortened daylight in winter stimulates melatonin secretion, which activates the reproductive hormone axis in the brain to release gonadotropin-releasing hormone. This, in turn, prompts the anterior pituitary gland to secrete follicle-stimulating hormone and luteinizing hormone, thereby triggering estrus behavior [11,12,13]. In contrast, high temperatures in summer can cause heat stress, which affects the secretion of gonadotropins [14,15,16], leading to less obvious estrus signs or even impaired reproductive performance.

In studies related to sexual maturity, conventional observational methods are reliable for determining the age of sexual maturity in boars. However, estrus signs in sows are often less pronounced. In addition to observation, methods such as hormone assays combined with ovarian ultrasound examination can be used to determine the estrus period. In the research by Antonio et al. [17], the accuracy of different methods for monitoring sow estrus was compared. It was found that ultrasound examination offers high accuracy but is heavily influenced by equipment and operator skill; progesterone concentration can serve as a marker for puberty, and models combining it with backfat thickness show good accuracy; external signs are easy to measure but are subjective and less accurate. In the study by L K G et al. [18], it was discovered that body weight at 75 days of age and vulva development changes between 95 and 115 days of age can assist producers in selecting replacement gilts, with vulva physiological development serving as a screening tool. With technological advancements, there are now methods for rapidly and accurately detecting estrus behavior in sows. In the research by Chenlei et al. [19], by collecting saliva from estrous sows and conducting proteomic analysis, potential salivary hormone proteins (GAPDH, CALR) were identified, proposing a new research direction for developing an estrus detection kit for sows. Zhang Zhen [20] proposed a machine vision-based method for estrus detection in sows, utilizing an improved contour recognition model and contour matching algorithm to achieve accurate detection of estrus. In the study by Zhuang Yanrong et al. [21], based on the characteristic of erect ears in *Yorkshire* pigs during estrus, a set of intelligent facilities for automatic estrus detection was developed using neural networks combined with machine learning. In the era of intelligent breeding with highly integrated computer technology, large-scale model algorithms, through multi-dimensional data fusion and deep learning mechanisms, are addressing technical challenges in traditional livestock production.

Research on foreign breeds has found that Danish *Yorkshire* pigs with early puberty exhibit better first-parity reproductive performance [22]. However, other studies analyzing the impact of puberty on reproductive performance in American *Yorkshire* pigs revealed that individuals with later puberty had higher first-parity litter sizes, possibly because sows with early puberty were not fully physically mature at first mating [23]. In the research by Gu Jianping et al. [24], reproductive traits over three parities were compared among *Taihu* pigs first mated at 4, 6, and 8 months of age. It was found that mating at 4 months did not result in significant differences in reproductive performance, but emphasized the need for enhanced feeding management. Similarly, in previous studies on local breeds, *Laiwu* pigs reached sexual maturity at 3–4 months, with first mating recommended at 6–8 months and a mating weight of 50 kg [25]; *Jinhua* pigs reached sexual maturity at 3.5 months, could be first mated at 5 months with a mating weight of 45 kg [26]; and *Min* pigs reached puberty at 112 days, with first mating recommended at 145 days and a mating weight of 60 kg [27]. In the study by Cottney [3], it was determined that mating at the third estrus cycle is optimal. In this study, when selecting pigs for first mating at 5 months of age, they were in their third or fourth estrus cycle; at 6 months, they were in their fifth or sixth estrus cycle. Based on this trial, the litter size, birth weight, weaning count, and weaning weight of sows mated at 5 months were lower than those mated at 6 months, but the differences were not statistically significant. Moreover, the survival rate was higher for sows mated at 5 months. Therefore, selection at 5 months of age is feasible. Boars and sows with a body weight of 60 kg and a backfat thickness of 18 mm meet the selection criteria, and sows should be first mated at or after their third estrus cycle.

### 3.2. Transcriptome Sequencing of Testicular Tissues from Shenxian Boars Before and After Sexual Maturity

Transcriptome sequencing was performed on testicular tissues of *Shenxian* pigs before and after sexual maturity, yielding a total of 11,033 differentially expressed genes, of which 6016 were up-regulated and 5017 were down-regulated. Through GO functional annotation and KEGG pathway analysis, it was found that the differentially expressed genes were mainly enriched in functional terms related to spermatogenesis, such as reproductive processes, sexual reproduction, ciliary movement, sperm flagella, and protease activation, as well as pathways associated with sexual maturation. Based on GO functional enrichment and KEGG pathway analysis, four candidate genes related to sexual maturity were identified: *TSSK6*, *SPATA16*, *CFAP43*, and *TEX101*. Previous studies have shown that *TSSK6* is a testis-specific kinase gene significantly positively correlated with high sperm motility and fertility. It is involved in the regulation of spermatogenesis and sperm function, is highly and specifically expressed in testicular tissues, and is associated with key processes of spermatogenesis [28]. Other research has indicated that the *TSSK6* gene may be related to the freeze-tolerance of boar semen [29]. The significant expression of the *TSSK6* gene in 6-month-old boars provides a theoretical basis for the subsequent cryopreservation of *Shenxian* boar semen. The primary function of *SPATA16* is acrosome formation and Golgi vesicle transport. It belongs to the SPATA family, which includes multiple members that are specifically expressed in testicular tissues and collectively regulate different stages of spermatogenesis. Previous studies have found that mutations in the *SPATA16* gene can lead to globozoospermia in male animals [30]. In a transcriptome sequencing study by GAO et al. [31] on bovine testicular tissues before and after sexual maturity, *SPATA16* was also identified as a candidate gene for sexual maturity. *CFAP43* is a key structural protein of cilia and sperm flagella, regulating flagellar assembly and movement. Mutations in *CFAP43* can cause flagellar dysfunction, leading to loss of sperm motility. Additionally, studies on sheep have found correlations between *CFAP43* and the prolificacy of Jining Grey goats [32] as well as body size traits of Shaanbei White Cashmere goats [33]. *TEX101* plays an important role in sperm development and spermatogenesis. It interacts with Ly6k to participate in the maturation of proteins required for sperm migration and sperm-oocyte interaction [34,35].

In previous studies, the *KISS* gene family and the *FSHR* gene have been extensively investigated as major genes related to sexual maturity. However, in this experiment, the expression level of the *KISS1* gene did not qualify it as a differentially expressed gene, while the expression levels of *KISS1R* and *FSHR* were too low and both showed down-regulation. Therefore, these prominent genes were not selected as candidate genes. The primary reason for this may be breed-specific differences. The four genes identified above are all related to sperm function. Since the hallmark of sexual maturity is the ability to produce sperm, these genes can be considered as influencing the sexual maturity of *Shenxian* pigs.

### 3.3. Transcriptome Sequencing of Testicular Tissues from Sexually Mature Shenxian Pigs and Shenxian × Large White Crossbred Pigs

Transcriptome sequencing of testicular tissues from sexually mature *Shenxian* pigs and their crossbreeds with *Yorkshire* pigs identified a total of 702 differentially expressed genes, of which 582 were up-regulated and 120 were down-regulated. GO functional enrichment analysis revealed significant associations with hormone metabolic processes, hormone biosynthesis, steroid metabolic processes, steroid biosynthesis, hormone level regulation, oxidoreductase activity, and related pathways. KEGG pathway analysis highlighted enrichment in steroid biosynthesis, cortisol synthesis and secretion, and ovarian steroidogenesis. From the differentially expressed genes, eight up-regulated genes—*INSL3*, *CYP11B2*, *SRD5A1*, *ARSE*, *AIG1*, *GPX1*, *ATG4A*, and *GUSB*—were selected as candidate genes related to sexual maturity in *Shenxian* pigs.

*INSL3* primarily regulates testicular development. Secreted by testicular Leydig cells, it plays a critical role in maintaining Leydig cell function and androgen production [36]. During the fetal stage, *INSL3* can be exchanged between fetuses via the allantoic cavity, leading to masculinization of female fetuses. Studies have also shown that *INSL3* acts as a paracrine factor in adult pig testes, binding to its receptor RXFP2 and influencing germ cells to regulate spermatogenesis [37]. *CYP11B2* is a steroid synthase belonging to the *CYP450s* (cytochrome P450 family), which extensively participates in the synthesis and metabolism of androgens and primarily functions in testicular Leydig cells [38]. Research has found that the formation of *CYP11B2* chimeric genes can cause 11β-hydroxylase deficiency (11β-OHD), subsequently leading to precocious puberty [39,40,41]. *SRD5A1* encodes steroid 5α-reductase type 1, an enzyme that catalyzes the conversion of testosterone into the more potent dihydrotestosterone (DHT). It plays a key role in regulating androgen metabolism, skin function, and immune responses. *SRD5A1* is a crucial catalytic enzyme for androstenone synthesis in pig adipose tissue, responsible for the final step of this hormone’s synthesis. Its transcriptional activity is significantly positively correlated with adipose androstenone concentration [42]. The *ARSE* gene encodes arylsulfatase E, primarily associated with chondrodysplasia punctata type 1 (*CDX1*). Located on the X chromosome, it is known to cause congenital skeletal and cartilage developmental disorders in males in humans [43]. In studies on bovine testicular tissues, the *ARSE* gene is often used as a specific marker for bovine testicular cells and is specifically expressed in immature Sertoli cells [44,45]. The *AIG1* gene, androgen-induced gene 1, belongs to the *AIG* family and is primarily involved in regulating androgen levels [46]. The *GPX1* gene encodes glutathione peroxidase 1, a selenium-rich protein that plays important roles in oxidative stress, glucose and lipid metabolism, and disease development [47]. In the study by Lee et al. [48], *GPX1* was classified as a key antioxidant gene. During fertilization with Landrace boar sperm, higher expression of the antioxidant gene *GPX1* in embryos helps reduce oxidative stress, thereby improving blastocyst formation rates. *ATG4A* is a cysteine peptidase related to autophagy, belonging to the *ATG4* family, which primarily regulates the lipidation modification of LC3 proteins [49]. Moreover, *ATG4A* is a key specific gene regulating autophagy and mitochondrial clearance during erythrocyte development [50]. In the research by Zhang Heng [51], the *ATG4A* gene plays a regulatory role in the 4-cell stage of early embryonic development in pigs. *GUSB*, beta-glucuronidase, is an important hydrolytic enzyme in lysosomes responsible for degrading glycosaminoglycans [52]. Shi Yuzhen [53] found that the *GUSB* gene has anti-PRRSV (porcine reproductive and respiratory syndrome virus) functions and is also considered a candidate gene for inguinal hernia.

The gene set identified in this section collectively forms a multi-level regulatory network driving early sexual maturity: At the hormonal level, *CYP11B2* and *SRD5A1* directly participate in steroid hormone synthesis and androgen activation, respectively, while *INSL3* regulates Leydig cell function, collectively contributing to the premature elevation of sex hormones. At the cellular structure and function level, *ARSE* and *ATG4A* support the specific testicular cellular environment and cellular autophagy clearance, respectively, providing the foundation for the premature development of germ cells. At the metabolic and stress response level, *GPX1* and *AIG1* synergistically regulate oxidative balance and lipid metabolism to cope with the metabolic demands of early gonadal activation. These genes interact systematically across three dimensions—hormonal signaling, cellular microenvironment, and metabolic adaptation—working in concert to promote premature gonadal maturation and the onset of early sexual maturity.

### 3.4. Genes for Sexual Precocity

While reviewing the literature, it is known that *KISS* is associated with the initiation of puberty [54,55]. However, in this experiment, the expression level of the *KISS* gene was not high, and thus it was not considered a candidate gene. In the study by Shi et al. [56], through cloning and analysis of the *Kiss1* gene, it was found that its expression gradually increased with age during testicular development in *Hezuo* pigs, playing an important regulatory role in boar puberty and spermatogenesis. In the research by M. J. F et al. [57], using gene editing technology to disrupt the *KISS* gene, it was discovered that even a small amount of functional *KISS* gene could support normal sexual development and maturation in pigs. In the study by Liu Ping et al. [58], the *Kiss-1* gene was found to play a significant role in the hypothalamic–pituitary–ovarian axis during pig puberty, with its expression changes closely related to reproductive hormone levels. Li Zhuozhao [59] found that while the *Kiss1* gene sequence was consistent between Meishan and Landrace × Yorkshire sows, its expression in the reproductive axis varied across breeds and ages, with high expression of *Kiss1* in the reproductive axis favoring the initiation of puberty in sows.

Apart from KISS-related genes, due to breed differences, various breeds exhibit different early sexual maturity genes. In Cao Haoming’s [60] study on *Hanjiang* black pigs, the *PAPPA2* gene was identified as influencing early sexual maturity in this breed. However, in this experiment, the expression level of the *PAPPA2* gene showed no significant difference and thus was not considered a candidate gene. In the research by Mo Jiayuan et al. [61] on *Guangxi* pigs, nine candidate genes related to reproductive traits and potentially influencing early sexual maturity were identified. In Chen Ye’s [62] study on *Taihu* pigs, the *GGA* gene was suggested as potentially influencing early sexual maturity in this breed. In the research by Mei Ziling et al. [63] on *Xiang* pigs, the *C1GALT1* gene was proposed as possibly influencing early sexual maturity in *Xiang* pigs.

In this experiment, based on the results of two transcriptome sequencing analyses, a total of 39 differentially expressed genes specifically expressed in *Shenxian* pigs were identified. Among these, seven had no functional annotations, while the remaining 32 were analyzed for their functions. From these, *SRD5A1* and *CYP11B2* were identified as genes that may affect precocious puberty in male pigs in *Shenxian* County. Existing research indicates that *SRD5A1* primarily catalyzes the conversion of testosterone into the more active dihydrotestosterone, playing a key role in regulating androgen metabolism [64]. Dysfunction or abnormalities in the *SRD5A1* gene can indeed lead to androgen disorders and even the onset of diseases [65]. This process clearly shows that *SRD5A1* influences early sexual maturity by activating testosterone and thereby affecting androgen levels. Moreover, in this experiment, the expression level of the *SRD5A1* gene in *Shenxian* pig individuals was consistently significant. Therefore, *SRD5A1* can be identified as a gene potentially influencing early sexual maturity in *Shenxian* pigs. *CYP11B2* belongs to the cytochrome P450 family, which is widely involved in the synthesis and metabolism of androgens. Due to its high homology with *CYP11B1*, unequal allelic exchange during meiosis can lead to the formation of *CYP11B2*/*CYP11B1* chimeric genes. In such chimeras, *CYP11B2* acts as a promoter and is not regulated by corticotropin-releasing hormone, resulting in cortisol deficiency and 11β-hydroxylase deficiency (11β-OHD), clinically manifested as early sexual maturity. Therefore, *CYP11B2* may also be considered a candidate gene influencing early sexual maturity in *Shenxian* pigs.

## 4. Materials and Methods

### 4.1. Phenotype Analysis Experiment

#### 4.1.1. Experimental Animals

Weaned *Shenxian* pig population.

#### 4.1.2. Experimental Design

Weaned *Shenxian* piglets were observed daily from 8:00 a.m. to 11:00 a.m. and from 2:00 p.m. to 5:00 p.m. for estrus behavior. For boars, the ages at which mounting, penile extension, and ejaculation occurred were recorded using the observational method. For sows, the onset and end times of the first, second, and third estrus cycles were recorded by combining the observational method, boar-teasing test, and back-pressure test. The duration of estrus and the interval between estrus cycles were also recorded. After all data were collected, sows in their third estrus cycle were selected for first mating. First-parity litter performance was recorded and compared with previous first-mating reproductive performance to determine whether significant differences existed, thereby assessing the feasibility of early mating. Body weight and backfat thickness at selection were recorded as selection indicators.

#### 4.1.3. Experimental Materials

Electronic cage scale, hanging scale, ultrasonic backfat instrument (Unicorn Vet, Advelma, Eijsden, The Netherlands), coupling agent, and clippers.

#### 4.1.4. Experimental Methods

The observational method was used to determine the timing of mounting, penile extension, and ejaculation behaviors in *Shenxian* boars. The age at which a boar could complete the entire sexual act and ejaculate was defined as the age of sexual maturity. For sows, the onset of estrus was determined by combining the observational method, boar-teasing test, and back-pressure test. After estrus was detected, the end time and duration of estrus were continuously monitored and recorded. The same procedure was used to record these data for the first three estrus cycles, and the interval between cycles was also noted. After observation, selection was carried out. Body weight was measured using an electronic cage scale, and backfat thickness was recorded with the backfat instrument to establish selection criteria. Sows in their third estrus cycle at selection were mated for the first time. After farrowing, the litter size, weaning count, and survival rate were recorded. The birth weight and weaning weight of piglets were measured using a hanging scale.

#### 4.1.5. Data Processing and Analysis

Excel 2016 was used for preliminary data processing and removal of outliers. SPSS 26.0 was used to perform independent-sample *t*-tests for litter size, litter weaning weight, weaning count, and litter birth weight. A chi-square test was applied to survival rate. Data are presented as mean ± standard deviation.

### 4.2. Molecular Experiment

#### 4.2.1. Experimental Materials

This experiment selected *Shenxian* boars from different litters provided by Hebei Zhengnong Agriculture Co., Ltd. (Shijiazhuang, China). The samples included three 1-month-old boars (designated as group S2), three 6-month-old boars (designated as group S1 in the first sequencing and as group S in the second sequencing), and three 6-month-old sexually mature *Shenxian* × Yorkshire crossbred boars (designated as group YS). Testicular tissues were collected from each boar by physical castration, rinsed with PBS buffer, placed in cryovials, rapidly frozen in liquid nitrogen, and transported on dry ice to a biotechnology company for RNA extraction and transcriptome sequencing.

First, differentially expressed genes were compared between groups S1 and S2. Subsequently, differentially expressed genes were compared between groups S and YS. Finally, the intersection of differentially expressed genes from both comparisons was analyzed for gene functions to screen candidate genes affecting early sexual maturity.

#### 4.2.2. RNA Extraction

Total RNA was extracted from tissue samples using the TRNzol method. After extraction, RNA concentration, RIN value, 28S/18S ratio, and fragment size were assessed using an Agilent 2100 Bioanalyzer (Santa Clara, CA, USA, Agilent Technologies, Inc.) to determine RNA integrity. RNA purity (OD260/280) was measured with a NanoDrop ultraviolet spectrophotometer (Waltham, MA, USA, Thermo Scientific).

#### 4.2.3. Library Construction, Sequencing, and Analysis

mRNA was enriched from total RNA using Oligo dT magnetic beads. After fragmentation, first-strand cDNA was synthesized with random hexamer primers, followed by second-strand cDNA synthesis. After end repair, A-tailing, adapter ligation, fragment selection, amplification, and purification, the libraries were ready. Qualified samples were sequenced on a DNBSEQ-T7 sequencer (Shenzhen, China, MGI, Inc.). Raw data were then quality-controlled and aligned to the reference genome. FPKM values for each gene were calculated based on gene length, and reads mapped to each gene were counted. Finally, differential expression analysis was performed, and GO functional enrichment and KEGG pathway analyses were conducted on the differentially expressed genes.

#### 4.2.4. q-PCR Validation

RNA was re-extracted from the same samples, and suitable differentially expressed genes were selected for quantitative real-time PCR analysis. The TPB gene was used as an internal reference, and the relative expression of target genes was calculated using the 2^−ΔΔCt^ method. The results were compared with the sequencing data for validation. The primer amplification information is shown in the table below (Table 10 and Table 11):

## 5. Conclusions

*Shenxian* boars reached sexual maturity at an average age of 116 days in winter and 129 days in summer. For sows, the first estrus occurred at 114 days, the second at 134 days, and the third at 154 days in winter; corresponding ages in summer were 125, 144, and 164 days. The duration of estrus was around 3 days, and the estrus interval was approximately 20 days for both seasons. The selection time for *Shenxian* pigs can be adjusted to 5 months of age, with selection criteria being body weight above 60 kg and backfat above 18 mm.

Through transcriptome sequencing of testicular tissue, SRD5A1 and CYP11B2 were identified as candidate genes that may affect precocious puberty in *Shenxian* pigs.

## Figures and Tables

**Table 1:** Comparison of Sexual Maturation Time (Age in Days) for *Shenxian* Boars in Winter and Summer.

Season	First Mounting	Penis Protrusion	Ejaculation
Winter	86.54 ± 10.74	102.10 ± 12.36	116.64 ± 11.99
Summer	88.74 ± 9.46	108.90 ± 9.78 **	129.60 ± 11.66 **
*p*-value	0.280	0.003	<0.001

Note: ** indicates extremely significant correlation (*p* < 0.01).

**Table 2:** Comparison of Sexual Maturation Time (Age in Days) for *Shenxian* Sows in Winter and Summer.

Estrus	Season	Estrus Onset	Estrus End	Duration (Days)	Estrus Interval (Days)
First	Winter	114.44 ± 4.82	116.76 ± 4.77	3.32 ± 0.55	-
	Summer	125.04 ± 4.25 **	127.02 ± 4.12 **	2.98 ± 0.51 **	-
	*p*-value	<0.001	<0.001	0.002	-
Second	Winter	134.72 ± 5.56	137.10 ± 5.49	3.38 ± 0.53	20.34 ± 1.74
	Summer	144.54 ± 4.46 **	146.66 ± 4.50 **	3.12 ± 0.48	19.50 ± 1.09 **
	*p*-value	<0.001	<0.001	0.12	0.009
Third	Winter	154.26 ± 6.69	1566.54 ± 6.73	3.28 ± 0.45	19.54 ± 1.59
	Summer	164.38 ± 4.14 **	166.50 ± 4.10 **	3.12 ± 0.48	19.84 ± 1.15
	*p*-value	<0.001	<0.001	0.090	0.283

Note: ** indicates extremely significant correlation (*p* < 0.01).

**Table 3:** Comparison of Sow Production Performance.

Group	Litter Size (Heads)	Birth Weight (kg)	Number Weaned (Heads)	Weaning Weight (kg)	Survival Rate (%)
C1 Group	9.75 ± 0.91	9.76 ± 0.78	9.40 ± 0.88	57.59 ± 5.56	96.53 ± 4.88
C2 Group	10.80 ± 1.32	10.37 ± 1.20	10.25 ± 1.12	61.32 ± 6.56	95.22 ± 5.81
*p*-value	0.06	0.65	0.11	0.60	0.27

**Table 4:** Selection Indicators.

Sex	Body Weight (kg)	Backfat (mm)
Boar	60.45 ± 3.79	16.82 ± 1.06
Sow	59.33 ± 2.69	16.18 ± 1.38

**Table 5:** Summary of Sequencing Data for S1, S2 Samples.

Sample	Raw Reads (G)	Raw Bases (G)	Clean Reads (M)	Clean Bases (G)	Q20 (%)	Q30 (%)	GC Content (%)
S1-1	44.24	6.64	44.21	6.61	97.72	93.44	47.98
S1-3	48.94	7.34	48.88	7.31	97.78	93.74	47.87
S1-5	49.81	7.47	49.76	7.45	97.49	92.86	48.43
S2-1	47.37	7.10	47.34	7.08	97.73	93.56	46.60
S2-3	56.76	8.51	56.72	8.49	98.07	94.43	46.18
S2-5	43.16	6.47	43.13	6.46	97.80	93.77	46.33

**Table 6:** Alignment Results of S1, S2 Samples to Reference Genome.

Sample	Total Clean Reads (G)	Total Mapped Rate	Uniquely Mapped Rate	Multi Mapped Rate	Mismatch Rate
S1-1	44.21	97.04%	92.37%	2.54%	2.13%
S1-3	48.88	96.13%	91.48%	2.49%	2.16%
S1-5	49.76	96.93%	92.02%	2.63%	2.28%
S2-1	47.34	96.81%	92.07%	2.38%	2.36%
S2-3	56.72	96.52%	92.18%	2.10%	2.24%
S2-5	43.13	97.06%	92.23%	2.40%	2.43%

**Table 7:** Summary of Sequencing Data for YS, S Samples.

Sample	Raw Reads (G)	Raw Bases (G)	Clean Reads (G)	Clean Bases (G)	Q20 (%)	Q30 (%)	GC Content (%)
YS1	48.43	7.27 G	46.83	7.03	99.38	97.67	50.07
YS2	42.42	6.36 G	40.96	6.14	99.37	97.67	49.88
YS3	44.69	6.7 G	42.98	6.45	99.36	97.61	50.47
S1	46.03	6.9 G	44.22	6.63	99.4	97.75	49.84
S2	45.67	6.85 G	43.98	6.6	99.36	97.59	49.8
S3	44.17	6.63 G	42.42	6.36	99.37	97.57	50.26

**Table 8:** Alignment Results of YS, S Samples to Reference Genome.

Sample	Total Clean Reads (G)	Total Mapped Rate	Uniquely Mapped Rate	Multi Mapped Rate	Mismatch Rate
YS1	46.83	97.79	95.04	2.75	6.67
YS2	40.96	97.4	94.83	2.58	7.15
YS3	42.98	97.58	94.88	2.7	7.02
S1	44.22	97.54	94.89	12.66	6.99
S2	43.98	97.54	94.88	2.66	7.03
S3	42.42	97.36	94.68	2.68	7.29

**Table 9:** Overlapping Genes from Both Sequencing Experiments.

Gene	NCBI Desc
*ALOX12*	arachidonate 12-lipoxygenase, 12S-type Encodes a lipoxygenase involved in arachidonic acid metabolism and inflammatory responses.
*AMBP*	protein AMBP; protein AMBP precursor Encodes the alpha-1-microglobulin/bikunin precursor protein, which possesses protease inhibitory and immunomodulatory functions.
*CAPN13*	calpain-13 Encodes calpain-13, a member of the calcium-dependent cysteine protease family.
*CPN2*	carboxypeptidase N subunit 2 Encodes a subunit of carboxypeptidase N, involved in degrading vasoactive peptides such as kinins to regulate blood pressure and inflammation.
*EPS8L3*	epidermal growth factor receptor kinase substrate 8-like protein 3 Encodes an epidermal growth factor receptor pathway substrate 8-like protein 3, participating in actin cytoskeleton remodeling and signal transduction.
*FBXL21*	LOW-QUALITY PROTEIN: F-box/LRR-repeat protein 21 Encodes F-box/LRR-repeat protein 21, a component of the SCF ubiquitin ligase complex, involved in the temporal regulation of protein degradation.
*GCM1*	chorion-specific transcription factor GCMa Encodes glial cells missing transcription factor 1, a key regulator of placental development.
*HCRTR2*	orexin receptor type 2 Encodes the orexin receptor type 2, involved in the sleep–wake cycle, energy homeostasis, and neuroendocrine regulation.
*IL1R2*	interleukin-1 receptor type 2;interleukin-1 receptor type 2 precursor Encodes interleukin-1 receptor type 2, which acts as a decoy receptor to inhibit IL-1-mediated inflammatory signaling.
*KLHL31*	kelch-like protein 31 Encodes Kelch-like protein 31, primarily expressed in skeletal muscle, with functions related to sarcomere structure maintenance.
*LCT*	LOW-QUALITY PROTEIN: lactase-phlorizin hydrolase Encodes lactase, responsible for lactose breakdown. Its persistent expression (adult lactase persistence) is associated with genetic variation.
*MC2R*	adrenocorticotropic hormone receptor Encodes the adrenocorticotropic hormone (ACTH) receptor, mediating ACTH-stimulated cortisol secretion from the adrenal cortex.
*NCAN*	neurocan core protein Encodes the neurocan proteoglycan, involved in cell adhesion and nervous system development.
*NTM*	neurotrimin Encodes neurotrimin, a cell adhesion molecule that functions in neural development and synaptic plasticity.
*SDSL*	serine dehydratase-like Encodes a serine dehydratase-like protein, potentially involved in serine metabolism.
*SMPD3*	sphingomyelin phosphodiesterase 3 Encodes neutral sphingomyelinase 2, which catalyzes sphingomyelin hydrolysis and is involved in apoptosis and lipid raft signaling.
*SNCG*	gamma-synuclein Encodes gamma-synuclein, associated with neuronal function and abnormally expressed in certain cancers.
*SRD5A1*	3-oxo-5-alpha-steroid 4-dehydrogenase 1 Encodes steroid 5-alpha-reductase 1, which catalyzes the conversion of testosterone to dihydrotestosterone (DHT), a key enzyme in androgen metabolism.
*SYTL3*	synaptotagmin-like protein 3 Encodes synaptotagmin-like protein 3, potentially involved in vesicle trafficking and secretion.
*TMC3*	transmembrane channel-like protein 3 Encodes transmembrane channel-like protein 3. Its function is not fully understood but may be related to sensory transduction.
*TNP1*	spermatid nuclear transition protein 1 Encodes transition protein 1, which replaces histones during spermatogenesis and is crucial for chromatin condensation.
*TRPC4*	short transient receptor potential channel 4 Encodes transient receptor potential cation channel subfamily C member 4, involved in calcium influx and various cellular signaling pathways.
*UABP-2*	uteroferrin-associated basic protein 2 precursor Acts as a progesterone-regulated uterine secretory protein responsible for transporting iron ions to the embryo during early pregnancy, supporting embryonic development and maintaining gestation.
*VIT*	vitrin Encodes vitellogenin, the main precursor protein of yolk in oviparous animals.
LOC100524382	LOW-QUALITY PROTEIN: L-amino-acid oxidase-like Involved in the oxidative metabolism of amino acids.
LOC100621771	arylsulfatase F-like Arylsulfatases participate in the hydrolysis of sulfate ester bonds and play roles in lysosomal function and signaling molecule regulation.
LOC100738548	LOW-QUALITY PROTEIN: Krueppel-like factor 17 The KLF family consists of zinc-finger transcription factors involved in various biological processes such as cell differentiation and proliferation.
LOC102157770	proteasomal ubiquitin receptor ADRM1 This protein is a component of the 26S proteasome, responsible for recognizing and binding polyubiquitinated proteins, directing them to the proteasome for degradation. It is a core regulator of proteostasis (protein homeostasis).
LOC102158087	-
LOC102161572	-
LOC102164954	-
LOC102165149	olfactory receptor 51F2-like Olfactory receptors belong to the G protein-coupled receptor superfamily, primarily mediating olfactory signal transduction.
LOC110259004	leucine-rich repeat-containing protein 37A-like Proteins containing leucine-rich repeats typically mediate protein–protein interactions and are involved in signal transduction or cell adhesion.
LOC110260194	cytochrome P450 11B2, mitochondrial Catalytic synthesis of aldosterone to regulate water salt balance
LOC110260274	-
LOC110260333	hydroxymethylglutaryl-CoA synthase, mitochondrial-like This is the rate-limiting enzyme in cholesterol synthesis (the mevalonate pathway). Cholesterol is the common precursor for all steroid hormones, so this enzyme’s function directly affects the synthetic capacity for steroid hormones.
LOC110260795	-
LOC110261213	-
LOC110261731	-

**Table 10:** Primer Sequence Information for q-PCR Amplification in S1, S2.

Gene	Forward Primer	Reverse Primer	Product Size (bp)	Annealing Temp (°C)
*TPB*	GATTGTGCCACAGCTGCAAA	CTCCCGTGCACACCATTTTC	191	60
*TSSK6*	TCAAGTGCGAAAACGTGCTG	GGTGCTTAGATCCGGGTAGC	101	60
*SPATA16*	CCTTTGCTCACCATGCCTCT	TCTGGTACACGCAAAGGGTC	179	60
*CFAP43*	TCTGCAGCTATCTTCTTCCTGA	CCCGGACACACAGAATTCCA	165	60
*TEX101*	ATCCTAGGAGCCCCAACCTT	GGCCAAAATTGCTGTCTCGG	195	60
*FOLR2*	GGAAGGGCACAACTCCCTC	CCTGGCTCTACCTTGTGGTG	144	60
*ITGA6*	GGGGCCCCACAGTATTTTGA	ATTTTTGACCGCAACGCCAA	188	60
*ERBB3*	CGGGGCTTCTCCTTGTTGAT	GCTGCCGATTGGCACTTATG	109	60
*SYT10*	ACCCGTGTACACCGAAAGAC	CCCTGGAGAGATCAGAGGCT	187	60

**Table 11:** Primer Sequence Information for q-PCR Amplification in S, YS.

Gene	Forward Primer	Reverse Primer	Product Size (bp)	Annealing Temp (°C)
*TPB*	GATTGTGCCACAGCTGCAAA	CTCCCGTGCACACCATTTTC	191	60
*INSL3*	TGCTACAGTGGCTGGAAGGACA	ACAGAGGGTCAGCAAGTCTTG	191	60
*CYP11B2*	CGTGTTCTTGCTAAACGGGC	AGATGCTGGGCTTGATGTCC	194	60
*SRD5A1*	CGCAAAGGGCTTTGGTCTTC	TGAACCACAAGGCGAAACCT	192	60
*ARSE*	ACCCACTCCGATCAGGTATGG	CAGTTGAGACCCAGATGCCAT	163	60
*AIG1*	TGGCTGAATCACGGAATGGG	TTTCTCTTCTTCCATACTTCTCTGT	199	60
*GPX1*	TGAATGGCGCAAATGCTCAC	ATTGCGACACACTGGAGACC	125	60
*ATG4A*	AGTTGAAGTTCGAGCGCAGT	TGTCATCCTGGGCCAATTCC	144	60
*GUSB*	CATCGATGAGAGTCCGGGTG	CTTGTCCCTGCGAACCATCT	106	60

## Data Availability

The original contributions presented in this study are included in the article. Further inquiries can be directed to the corresponding author.
